# Toward a More Complete, Flexible, and Safer Speed Planning for Autonomous Driving via Convex Optimization

**DOI:** 10.3390/s18072185

**Published:** 2018-07-06

**Authors:** Yu Zhang, Huiyan Chen, Steven L. Waslander, Tian Yang, Sheng Zhang, Guangming Xiong, Kai Liu

**Affiliations:** 1 School of Mechanical Engineering, Beijing Institute of Technology, Beijing 100081, China; yu.zhang.bit@gmail.com (Y.Z.); chen_h_y@263.net (H.C.); ytiangsky@gmail.com (T.Y.); zhanggugeguge@gmail.com (S.Z.); leoking1025@gmail.com (K.L.); 2 University of Toronto Institute of Aerospace Studies, Toronto, ON M3H 5T6, Canada; stevenw@utias.utoronto.ca

**Keywords:** speed planning, convex optimisation, autonomous driving, friction circle, driving safety, dynamic obstacle avoidance, ride comfort, mobility

## Abstract

In this paper, we present a complete, flexible and safe convex-optimization-based method to solve speed planning problems over a fixed path for autonomous driving in both static and dynamic environments. Our contributions are five fold. First, we summarize the most common constraints raised in various autonomous driving scenarios as the requirements for speed planner developments and metrics to measure the capacity of existing speed planners roughly for autonomous driving. Second, we introduce a more general, flexible and complete speed planning mathematical model including all the summarized constraints compared to the state-of-the-art speed planners, which addresses limitations of existing methods and is able to provide smooth, safety-guaranteed, dynamic-feasible, and time-efficient speed profiles. Third, we emphasize comfort while guaranteeing fundamental motion safety without sacrificing the mobility of cars by treating the comfort box constraint as a semi-hard constraint in optimization via slack variables and penalty functions, which distinguishes our method from existing ones. Fourth, we demonstrate that our problem preserves convexity with the added constraints, thus global optimality of solutions is guaranteed. Fifth, we showcase how our formulation can be used in various autonomous driving scenarios by providing several challenging case studies in both static and dynamic environments. A range of numerical experiments and challenging realistic speed planning case studies have depicted that the proposed method outperforms existing speed planners for autonomous driving in terms of constraint type covered, optimality, safety, mobility and flexibility.

## 1. Introduction

Speed planning plays an important role in guaranteeing the ride comfort and safety in autonomous driving applications. All different kind of scenarios together raises distinct requirements and consequently different constraint types for speed planning problem formulations, which makes it challenging to solve.

In most of urban driving scenarios, autonomous driving systems prefer smooth speed profiles for the sake of ride comfort. These scenarios require the speed planner to consider the maximum lateral and longitudinal accelerations and decelerations (comfort box (CB) constraints), jerk (smoothness (S)) to manage smooth transitions between states of cars from time to time. Such a smooth speed profile with these bounds does not only exhibit energy-saving behaviors of autonomous cars but also presents a decent reference that is easy to track for a speed controller, which results in a pleased ride experience for passengers in the end.

There are some scenarios that need the speed planner to exploit the full mobility capacity of cars such as driving on the limits to pursue high speeds or dealing with emergencies [1]. These applications raise a common hard constraint called friction circle (FC) constraint that is related to vehicle dynamics and road conditions and a soft constraint called time efficiency (TE). Both constraints are closely related since the time efficiency objective will push a car to the limits to achieve the minimum travelling time, which may frequently activate the friction circle hard constraint during planning. A typical example is that cars race in a prescribed curvy track for speed. In academia, a large body of research is carried out to address the minimum-time speed planning problem over a fixed path and the outstanding ones of them are [2,3], which inspire our work in this paper.

Although constantly pursuing high speeds is not the goal of autonomous driving, varying weather conditions may have an impact on the road quality in such a way as to dramatically reduce the friction coefficients and in consequence the maximum safe velocity limits for vehicles [4]. Thanks to the rapid development of mature sensing, perception and scene understanding system relying on computer vision and machine learning techniques for autonomous driving, high level information such as weather conditions, road surface categories, together with vehicle state information, can be delivered from on-board perception systems to road friction estimators [5,6]. The environment-dependent, varying friction coefficient becomes available to speed or motion planners online, which can be used to generate safety-guaranteed speed profiles. Above applications all require the speed planning to consider the friction circle constraints in the problem formulation explicitly. Unfortunately, most of existing speed planner [7,8,9,10,11] does not take it into consideration. They conservatively search for solutions in a subset of the friction circle region, which sacrifices the mobility for safety. In addition, speed planning is oftentimes the last action to guarantee safety by regulating the speed to stop the car in front of obstacles when there is no room to adjust the shape of the path to avoid collision in emergencies. This imposes a zero speed constraint at the end of the path, which is called boundary condition (BC) constraints in this paper. The lack of this kind of constraint in [3,11] produces flaws in safety in their planners.

In dynamic environments, speed planning also makes a difference in terms of dynamic obstacle avoidance. Regulating speed along the fixed path to avoid dynamic obstacles rather than swerving the path to deal with the dynamic obstacles (such as pedestrians, cyclists who are crossing lanes, changing lanes or turning in the intersection) may be thought of as a smart, energy-saving, and risk-free behavior in certain situations. It also should be noted that not all the cases with dynamic obstacles can be overcome using this method. It works under the the assumption that the path has been well predefined using some sampling techniques. Given the prescribed path, overtaking a slow front car using an opposite lane [11] imposes one or several time window (TW) constraints for speed planning in time domain at the conflict region with other road participants along the path. Merging from a freeway entrance ramp to a lane with an oncoming high-speed vehicle on expressway [12,13] does not only bring in a time window constraint but also boundary conditions such as a desired final speed and acceleration constraints to keep the pace with other traffic participants. These cases require that the car reaches a certain point on the path in the time window to avoid collision. However, most of the existing methods [3,4,7,8,14] ignore these constraints, which make their methods applicable only in static environments.

From the task perspective, the speed limit traffic sign along a road enforces a speed limit on a certain segment of a path, which is known as a path constraint (PC), that is, a hard constraint, in optimal control domain. In the case that desired speed profile is given by high level modules such as behavior planners or task planners, the integral of deviations (IoD) between planned speeds and desired speeds over the path is used as an objective to optimized to accomplish certain goals, which is a soft constraint.

All aforementioned constraints are summarized and categorized by us in Table 1, as *requirements* for the speed planning module to meet and *metrics* to identify the capacity of speed planners. Please note that we introduce a “*semi-hard*” constraint type to describe the unique characters of comfort box (CB) constraints. By “*semi-hard*”, we mean this kind of constraints should be satisfied in the first place to achieve high performance when possible and can be violated to meet fundamental motion safety requirements while penalizing violations. This is different from soft constraints that treat all the solution space equally by assigning the same coefficients to the soft constraints.

A **safety-guaranteed speed planner** should be able to generate a solution satisfying at least all the hard constraints (safety) in Table 1. A mature speed planner should cover all the constraints that include soft and hard ones.

By taking some additional steps beyond the seminal work done by [2,3], we present a general speed planning framework specifically for autonomous driving that is able to handle a wide range of different scenarios using convex optimization subject to a large collection of relevant constraints. Our contributions are as follows:

- We summarize the most common constraints raised in various autonomous driving scenarios as the *requirements* for speed planner design and *metrics* to measure the capacity of the existing speed planners roughly for autonomous driving. We clarify which constraints need to be addressed by speed planners to guarantee safety in general.
- In light of these *requirements* and *metrics*, we present a more general, flexible and complete speed planning mathematical model including friction circle, dynamics, smoothness, time efficiency, time window, ride comfort, IoD, path and boundary conditions constraints compared to similar methods explained in [3,11]. We addressed the limitations of the method of Lipp et al. [3] by introducing a *pseudo jerk* objective in longitudinal dimension to improve smoothness, adding time window constraints at certain point of the path to avoid dynamics obstacles, capping a path constraint (most-likely non-smooth) on speed decision variables to deal with task constraints like speed limits, imposing a boundary condition at the end point of the path to guarantee safety for precise stop or merging scenarios. Compared to the approach of Liu et al. [11], our formulation optimizes the time efficiency directly while still staying inside of the friction circle, which ensures our method exploits the full acceleration capacity of the vehicle when necessary.
- We introduce a semi-hard constraint concept to describe unique characters of the comfort box constraints and implement this kind of constraints using slack variables and penalty functions, which emphasizes comfort while guaranteeing fundamental motion safety without sacrificing the mobility of cars. To the best of our knowledge, none of the existing methods handle these constraints like ours. In contrast, Refs. [7,8,9,10,11] regarded comfort box constraints as hard constraints, which dramatically reduces the solution space and in consequence limits the mobility of cars.
- We demonstrate that our problem still preserves convexity with the added constraints, and hence, that the global optimality is guaranteed. This means our problem can be solved using state-of-the-art convex optimization solvers efficiently as well. We also provide some evidence to prove that our solution is able to keep consistent when the boundary conditions encounter some disturbances, which means only the part of results needed to be adjusted will be regulated due to the global optimality. This may benefit the track performance of speed controllers by providing a relative stable reference. It is not the case for these methods that solve the speed planning problem using local optimization techniques like [11]. A small change of boundary conditions or initial guess may result in a totally different solution due to local minimas in their problem.
- We showcase how our formulation can be used in various autonomous driving scenarios by providing several challenging case studies solved in our framework, such as safe stop on a curvy road with different entry speeds, dealing with jaywalking in two different ways and merging from a freeway entrance ramp to expressways with safety guaranteed.

This paper is organized as follows. Section 2 reviews the featured speed planning methods for autonomous driving. Section 3 formulates the problem for speed planning along a fixed path by considering different constraints. Section 4 describes the implementation details. Section 5 shows a rich set of numerical experiment results and Section 6 demonstrates three case studies with parameters from real platforms. Section 7 draws conclusions.

## 2. Related Work

A rich literature exists on speed planning as a single research topic or part of motion planning systems. Speed planning methods used in literature fall into two categories roughly: coupled speed planning and decoupled speed planning. The former family exists in motion planning frameworks that explore the spatial-temporal space simultaneously using optimization techniques [15,16,17] or search algorithms [18,19]. Most of the time-parameterized trajectory planning based on optimal control belong to this family. Due to the non-convexity of objectives, dynamics and other constraints, it is already very hard to find a feasible path, let alone a time optimal trajectory. Finding a time optimal path may take a lot of time. Thus it is impracticable to apply these methods to autonomous driving applications due to run-time requirements. The latter family frequently appears in hierarchical motion planning frameworks [9,20,21,22] that decouple motions by planning a path first then reconstructing a speed profile along the path, or shows up as a standalone research with the assumption that the path is known.

As we focus on the second class of the problem, we review these featured methods that are closely related to ours, which generate speed profiles along a fixed path subject to certain constraints. We first compare them with others in terms of constraints coverage, optimality, safety, flexibility, and capacity without revealing details, as seen in Table 2. Most of the existing methods just provided a workable speed profile rather than an optimal one for autonomous driving. None of them covered all the constraints we list in Table 1.

Li et al. [7] employed a trapezoidal speed profile with constant accelerations and decelerations along the fixed path in a hierarchical trajectory planning framework and smoothed the ramp-up and ramp-down part of trapezoidal speed profiles with 3rd-order polynomials, which is neither optimal nor flexible. Besides, the acceleration value may exceed the threshold after smoothing. Thus very conservative accelerations for the ramp-up and deceleration for the ramp-down were selected in their work. Most of the constraints in Table 1 were not covered in their speed planner.

**Figure 1:**
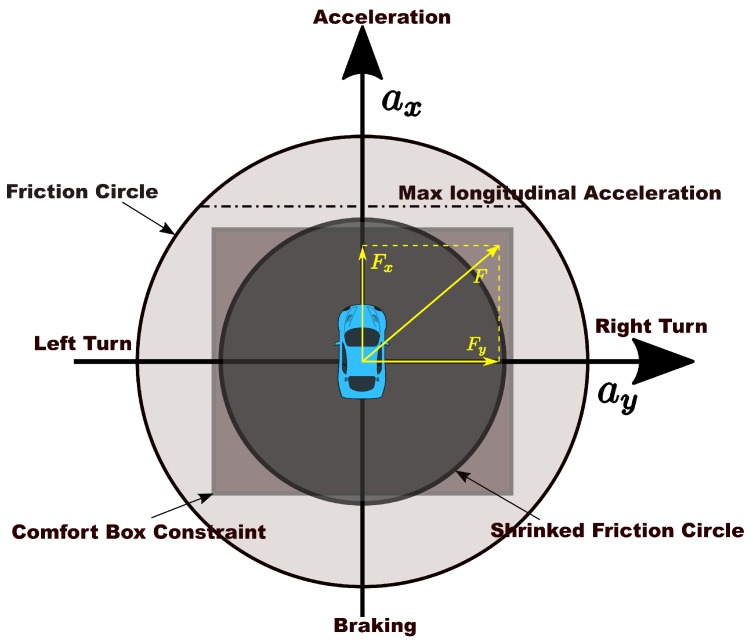
The comparison of solution spaces of the normal friction circle, the comfort box and the shrinked friction circle constraints.

Gu et al. [8] developed a constraint-based speed planner that trimmed the reference speed profile according to maximum velocity, lateral acceleration, longitudinal acceleration and deceleration. Since smoothness of the speed profile is not taken into account, the excessive longitudinal jerk may be observed [9]. They considered moving obstacles in speed planning in a reactive way based on the distance between obstacles and the ego car to affect speed profiles in the following work [10] and further enforced jerk limits on the speed profile in [9] for the sake of smoothness. As dynamics constraints are considered separately in the form of comfort box constraints that its upper boundaries need to be selected conservatively to prevent the total force from exceeding the friction force limits, the capacity of driving on the limits to deal with emergencies or pursue time efficiency is highly restricted. The difference of potential solution space of comfort box constraints and friction circle constraints is shown in Figure 1. In addition, the reduction of friction coefficient in extreme weather conditions will shrink the friction circle and the original fixed comfort constraints may create one or several dangerous zones in solution space, as shown in Figure 1, which will inevitably cause potential safety issues.

Dakibay et al. [4] exploited an aggressive speed planning method by numerically solving a nonlinear differential equation (NDE) about friction circle constraints and capping the speed profile with forward and reverse integration of accelerations results along the fixed path. Due to the approximation of solution of NDE, the full capacity of car is not explored. None of their results reaches exactly the friction circle. As the driving conditions are quite close to the limits, admissible room left for track errors is little. We argue that the smoothness of speed profiles still need to be considered to improve tracking performance of the controller for safety concerns (jerky speed profiles may result in overshooting and oscillation of controllers), even for aggressive driving scenarios, which did not appear in their solution.

Lipp et al. [3] presented a convex-optimization-based general minimum time speed planning method over the fixed path based on the approach proposed by [2]. The friction circle constraint is well considered as a convex set constraint acting on the problem formulation, which leads to an elegant solution. Not only the capacity of mobility of cars are fully explored, but also the total time travelling along the path is explicitly and analytically represented as a soft constraint to achieve time efficiency. The problem is solved by a customized interior point method using log barrier functions efficiently. Thanks to the preserving convexity of the problem formulation, the global optimality of solutions is guaranteed. However, smoothness of the speed profile is not consider, which most likely results in the same issues that we mentioned about Dakibay’s work regarding tracking performance and safety concerns. In addition, the use of customized Newton-based solver requires that constraints and objective functions are all at least twice differentiable, which seems very restrictive on the type of constraints that users can impose in convex optimization. Convex problems with non-differentiable constraint terms cannot be solved by their framework.

Liu et al. [11] recently introduced a temporal optimization approach, optimizing time stamps for all waypoints along a fixed path with respect to time window constraints at each point, and then using a slack convex feasible set algorithm to solve it iteratively. Smoothness of the speed profile and time efficiency are taken into account in the problem formulation. However, the time efficiency is considered in an indirect way that optimizes IoD with respect to a reference speed over the path. Their formulation leads to a highly nonlinear and non-convex problem and is solved by a local optimization method, thus only local optimality is guaranteed. They addressed some important constraints in Table 1 such as smoothness, time window and comfort box constraints in their formulation but left out the friction circle constraint, which does not fully exploit the acceleration capacity of the vehicle. In addition, since they optimized timestamps directly, we do not see a quick way to impose a path constraint or a point constraint as a hard one to manipulate speed profiles.

## 3. Problem Formulation

Assuming a curvature continuous path has been generated by a hierarchical motion planning framework like [9,22], the speed planning is to find a time-efficient, safe, and smooth speed profile travelling along the fixed path with respect to both safety and performance constraints.

To solve the proposed problem, we optimize the performance criterions from three aspects, smoothness $J_{S}$, time efficiency $J_{T}$, and speed deviation $J_{V}$ from a desired speed, with others left as hard constraints or semi-hard constraints. We first introduce the path representation and explain the relationship of an arc-length parametrized path and a time parametrized path, then present mathematical expressions of all the constraints, and pose the optimization problem at the end.

### 3.1. Path Representation

The goal of speed planning is to find a speed profile along a fixed path. Since the path is known, we need to reconstruct the mapping between the known path and the speed profile, then represent the speed profile with parameters determined by the prescribed path. A rich set of parameterized path representations has been proposed in the literature, including B-spline [23,24], Bezier curve [25,26], clothoid [27,28], polynomial curve [29] and polynomial spiral [30,31]. It is trivial to convert all the listed curve models to a simple waypoints representation, but not vice versa. To avoid the non-trivial converting between curve models, we use the general waypoints parametrization to represent a fixed path, with the orientation and curvature encoded implicitly by the path. Formally, we define a waypoints parametrized curve as a workspace path. A workspace path, *r*, of the body point, *b*, at the center of the rear axle with footprint, A, is defined as $r:\left[0,s_{f}\right]\to R^{2}$. More specifically, we consider the following arc-length parametric form in Cartesian coordinate system,

(1) $$\begin{array}{c} r\left(s\right)=\left(x\left(s\right),y\left(s\right)\right),s\in \left[0,s_{f}\right], \end{array}$$

where *s* is the arc-length parameter along the path, x(s) and y(s) are the scalars along two orthogonal base axes respectively. The relationship between the arclength *s* and the corresponding time *t* is formed as the function s=f(t), therefore the time parameterized workspace path $\tilde{r}\left(t\right)=\left(\tilde{x}\left(t\right),\tilde{y}\left(t\right)\right),t\in \left[0,t_{f}\right]$ can be easily acquired by substituting in for *s*.

Since the path, r(s), is known, the speed vector $\vec{v}$ in Cartesian coordinates can be calculated as below (the prime ′ and the dot · denote derivatives with respect to the arc-length, *s*, and the time, *t*, respectively for a curve throughout the paper),

(2) $$\begin{array}{c} \vec{v}=\dot{r}\left(s\right)=r^{\prime }\left(s\right)\dot{f}, \end{array}$$

where $r^{\prime }\left(s\right)$ is the unit tangent vector of the path r(s) at *s* that represents the direction of the speed of a car by assuming no sliding, $\dot{f}$ is the corresponding longitudinal speed of the car in ego frame. Let θ(s) represent the heading of the car at *s* of the path *r*, we get

(3) $$\begin{array}{c} r^{\prime }\left(s\right)=\left(\cos\left(\theta (s)\right),\sin\left(\theta (s)\right)\right)=\left(x^{\prime }\left(s\right),y^{\prime }\left(s\right)\right). \end{array}$$

The acceleration vector $\vec{a}$ in Cartesian coordinates system is

(4) $$\begin{array}{c} \vec{a}=\ddot{r}\left(s\right)=r^{\prime \prime }\left(s\right){\dot{f}}^{2}+r^{\prime }\ddot{f}, \end{array}$$

where $\ddot{f}$ is the longitudinal acceleration and $r^{\prime \prime }\left(s\right)$ is the principal normal vector of the path, which is also called the curvature vector. The 2-norm of the $r^{\prime \prime }\left(s\right)$ is the scalar of the curvature

(5) $$\begin{array}{c} \kappa =∥r^{\prime \prime }∥. \end{array}$$

### 3.2. Vehicle Model and Vehicle Dynamics Constraints

**Figure 2:**
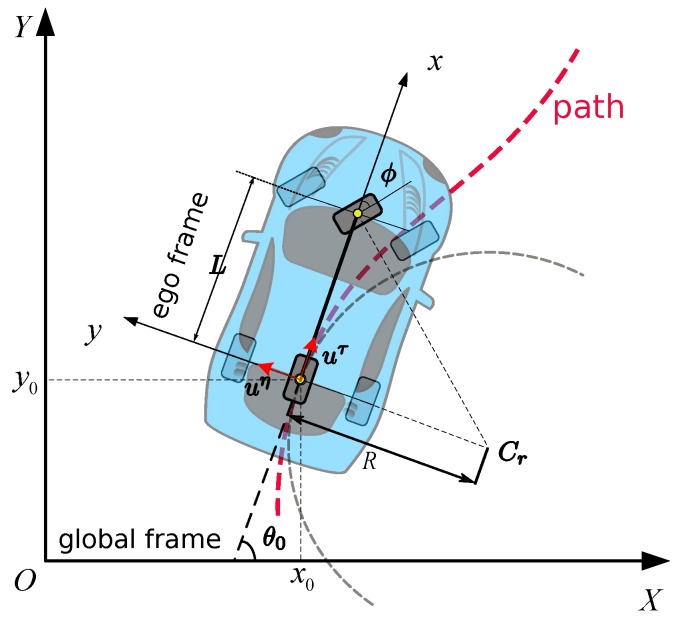
Single track car model.

Due to the non-holonomic dynamics of the vehicle system, the lateral motion and longitudinal motion are intrinsically coupled in a way that the car cannot move laterally without longitudinal speeds. The lateral motion is explicitly expressed by the prescribed path. The longitudinal motion is the goal of this paper. To build the connection between them and describe the vehicle dynamics explicitly in the problem formulation, we employ the single track vehicle model [32] (see Figure 2) to represent the actual vehicle kinematics and dynamics, which is widely used in motion planning research [9,19,22,30] and performs satisfactorily in practice [33]. The control force is defined as $u=(u^{\tau },u^{\eta })$, where $u^{\eta }$ is the lateral force and $u^{\tau }$ is the longitudinal force in ego frame. The dynamics of the car are given by

(6) $$\begin{array}{c} Ru=m\ddot{r}, \end{array}$$

where $R=\left[\begin{array}{cc} \cos(\theta (s)) & -\sin(\theta (s))\\ \sin(\theta (s)) & \cos(\theta (s)) \end{array}\right]$ is the rotation matrix that maps forces from the ego frame to the global Cartesian coordinate system, *m* is the mass of the car. We replace the $\ddot{f}$ with a function α(s), ${\dot{f}}^{2}$ with a function β(s) according to [2],

(7) $$\begin{array}{c} \alpha \left(s\right)=\ddot{f},\beta \left(s\right)={\dot{f}}^{2}. \end{array}$$

Then, $\dot{\beta }\left(s\right)=2\ddot{f}\dot{f}=2\alpha \left(s\right)\dot{f}=\beta^{\prime }\dot{f}$. Thus,

(8) $$\begin{array}{c} \beta^{\prime }\left(s\right)=2\alpha \left(s\right),s\in \left[0,s_{f}\right]. \end{array}$$

Therefore, Equations (4), (6) and (8) form the dynamics constraints of cars.

### 3.3. Friction Circle Constraints

Given sufficient engine powers, it is well known that the traction power of the car produced by tires to drive the car is limited by frictions between tires and the road surface. The combination of lateral and longitudinal control forces that is able to be leveraged by cars should stay inside a friction circle to prevent slipping or car from running out of control, which is defined as below

(9) $$\begin{array}{c} ∥u∥\leq \mu mg, \end{array}$$

where μ is the coefficient of friction between the tires and the road surface. The longitudinal force upper boundary can be calculated according to the maximum longitudinal acceleration by $u^{\tau }\leq m\cdot a_{max}^{\tau }$. This is only a necessary condition but not a sufficient condition to limit decision variables within the physical limits such as the nominal power. Take a driving case along a straight line for example, the speed will constantly increases to infinity if a fixed longitudinal force acts on the car and the path is long enough. However, in reality, the max force that a plant system can provide is also limited by the nominal power of the engine. For most of the time, the actual power used by car systems is maintained below the nominal power *P*, shown as below,

(10) $$\begin{array}{c} u^{\tau }\dot{f}\leq P, \end{array}$$

which also means, if the nominal power is reached, the driving force that a car is able to provide will decrease when the speed increases. This constraint is obviously nonlinear and non-convex. This issue ignored by [3] was first pointed out by Zhu et al. [20], but they did not solve it and left it as future work. Here we provide our solution by adding an upper boundary constraint on speed profiles according to platform limits. It will prevent the speed from increasing without limits. Other constraints like path constraints, boundary condition constraints, and the smoothness objective will also restrict the upper boundary of speed profiles. By doing so, we partially address this issue without bringing in non-convexity to our problem formulation. Given these factors, the formal mathematical representation of friction circle constraints can be defined as below,

(11) $$\begin{array}{cc} \left(\alpha (s),\beta (s),u(s)\right)\in & \left\{\left(\ddot{r}\left(s\right),{\dot{r}}^{2}\left(s\right),u\left(s\right)\right)\;\right|\\ & \;∥u(s)∥\leq \mu mg,\\ & \;u^{\tau }\left(s\right)\leq m\cdot a_{max}^{\tau },\\ & \;\beta \left(s\right)\leq v_{max}^{2}\}. \end{array}$$

### 3.4. Time Efficiency Objective

Different from the approach used in [11] that optimizes deviation between the planned speed and desired speed to ensuring time efficiency implicitly, we optimize the total traveling time along the fixed path from 0 to $s_{f}$ directly like [2,3], which can be expressed as $J_{T}=T=\int_{0}^{T}1dt$. Substitute the time variable *t* with arclength *s* and we get

(12) $$\begin{array}{c} J_{T}=T=\int_{f(0)}^{f(t_{f})}\frac{1}{\dot{f}}ds=\int_{0}^{s_{f}}\beta {\left(s\right)}^{-\frac{1}{2}}ds. \end{array}$$

### 3.5. IoD Objective

In autonomous driving applications, users, a behavior planning module or a task planning module may assign a reference speed $v_{r}\left(s\right)$ profile for a car to track. It is not a strict constraint like max speed thresholds or speed limits on the road that cannot be exceeded. Thus we introduce the integral of deviations between the planned speed and desired speed over the path as a soft constraint to measure this kind of performance, expressed as follows,

(13) $$J_{V}=\int_{0}^{s_{f}}∥\beta \left(s\right)-v_{r}{\left(s\right)}^{2}∥ds.$$

Unlike Ref. [11] regarding it as the measurement of time efficiency, we call it the task soft constraint, which makes more sense according to the purpose it serves in the form of (13).

### 3.6. Smoothness Objective

Direct tracking of a minimum-time speed profile will lead to joint vibrations and overshoot of the nominal torque or force limits of actuators [34,35]. When this happens in autonomous driving cars, it most likely results in bad ride experience and unstable driving behaviors. To ensure a smooth speed profile for better tracking performance, reducing wear of power train systems and guaranteeing the ride comfort at the same time, the smoothness of the trajectory needs to be considered. Since we assume a smooth and curvature-continuous path has been generated by a path planning module, we only consider the longitudinal jerk component of the trajectory. Formally speaking, jerk is the first derivative of acceleration in terms of time *t*, which also means the second derivative of velocity and the third derivative of position. According to (7) and (8), the jerk J(s) of the speed profile can be calculated as follows,

(14) $$\begin{array}{cc} J(s) & =\dddot{f}=\dot{\alpha }\left(s\right)=\alpha^{\prime }\left(s\right)\dot{f}\\ & =\alpha^{\prime }\left(s\right)\sqrt{\beta (s)}=\frac{1}{2}\beta^{\prime \prime }\left(s\right)\sqrt{\beta (s)}, \end{array}$$

which is nonlinear and non-convex. In fact, various smoothness metrics, including jerk, have been proposed to quantify the motion smoothness in literature [36,37]. However, the jerk objective brings in non-linearity and non-convexity, which makes our problem hard to solve, a better measurement which covers all the aspects we care about and also with good mathematical properties should be selected for the sake of fast convergence rate and optimality. Therefore, we introduce a *pseudo jerk* $\alpha^{\prime }\left(s\right)$, which is the first derivative of acceleration with respect to the parameter arc-length *s*, to the problem to encourage smooth transitions between states. The smoothness objective is then defined as

(15) $$\begin{array}{c} J_{S}=\int_{0}^{s_{f}}{∥\alpha^{\prime }\left(s\right)∥}^{2}ds, \end{array}$$

which is convex. By minimizing the variation of acceleration in terms of parameter *s*, a smooth acceleration profile is preferred. By integrating the smooth acceleration along *s*, the speed profile can be further smoothed.

### 3.7. Path Constraints

Path constraints can be defined as the following form,

(16) $$\begin{array}{c} \psi \left(s,x,u\right)\leq 0,\forall s\in \left[0,s_{f}\right], \end{array}$$

where *s* is arclength or time, *x* is the state of the system and *u* is the control variable. It restricts the range of values of states or controls, or the mixed one of both over the time or arc-length interval, or sub-interval of either for safety reasons or task requirements [38]. The rationales behind imposing these constraints in our problem are:

- Speed limits on certain segments of roads happen to be common driving scenarios in urban environments. The speed limits cannot be exceeded by autonomous driving systems, or the driving system will violate the traffic regulations and be fined. The restrictions may happen along the whole path or just segments of the path, which is a little different from an overall speed threshold constraint and the IoD objective.
- A high-level planning system (i.e., behavior planning system, task planning system) may provide the upper boundary or lower boundary of the speed profile to a speed planner to make it behave well or satisfy certain task requirements. A speed planner has to plan a speed profile that stays in the prescribed region or below the envelope.

**Figure 3:**
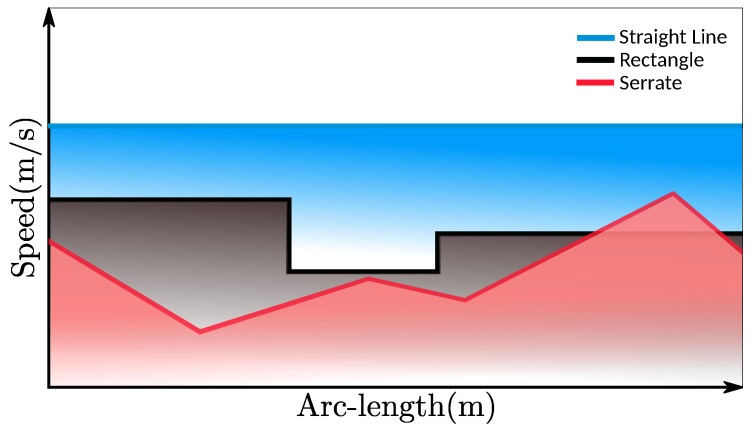
Different shapes of path constraints.

Both cases enforce hard constraints on speed profiles (state), which cannot be ensured by using soft constraints of speed deviation presented in [11] or the IoD constraint described by us. The residues in soft constraint form can be minimized by optimization, but how the state (velocity) approaches the reference is not determined. Overshooting or oscillation may occur around the reference during the optimization. However, a hard constraint like (16) is able to limit the “trace” of the system states strictly. More concisely, the specific constraints in our problem are expressed in the following form without involving control variables explicitly,

(17) $$\begin{array}{c} \beta \left(s_{i}\right)\leq \bar{\beta }\left(s_{i}\right),\forall s_{i}\in \left[s_{m},s_{n}\right], \end{array}$$

where $\bar{\beta }$ is the upper boundary of β at *s*, $0\leq s_{m}\leq s_{n}\leq s_{f}$ and m<n. Three typical path constraints shapes of $\bar{\beta }\left(s_{i}\right)$ are demonstrated in Figure 3.

### 3.8. Boundary Condition Constraints

The boundary condition constraints specifically refer to the terminal constraints that can be generally represented by

(18) $$\begin{array}{c} g(s_{f},x_{f},u_{f})\leq 0, \end{array}$$

where $x_{f}$ is terminal state variable and $u_{f}$ is the final control variable. More specifically, we impose the following constraint type,

(19) $$\begin{array}{c} {\underline{\alpha }}_{s_{f}}\leq \alpha_{s_{f}}\leq {\bar{\alpha }}_{s_{f}}\\ {\underline{\beta }}_{s_{f}}\leq \beta_{s_{f}}\leq {\bar{\beta }}_{s_{f}}. \end{array}$$

With ${\underline{\alpha }}_{s_{f}}\leq {\bar{\alpha }}_{s_{f}}$ and ${\underline{\beta }}_{s_{f}}\leq {\bar{\beta }}_{s_{f}}$, we can enforce either equality constraints (by “=”) or target set inequality constraints (by “<”) on the terminal state of the speed profile. These constraints involve two types of typical applications. One is the scenario that the car needs to fully stop in front of obstacle at a certain point on the path or at the end of the path. A zero speed and a zero acceleration at $s_{f}$ need to be guaranteed in this case. The other scenario occurs as a car tries to merge into an expressway from an entrance ramp, which needs to have the final speed fall in the speed limit range of the expressway. Other applications, such as keeping a fixed distance to the front car at the end of the path while matching the final speed with that of the front car can also be solved using this constraint in our framework. Such capacities are not present in [3,11]. If no strict boundary conditions on terminal states are required, the constraints can be deactivated by making ${\underline{\alpha }}_{s_{f}}=-\mu g$, ${\underline{\beta }}_{s_{f}}=0$, ${\bar{\alpha }}_{s_{f}}=\mu g$, ${\bar{\beta }}_{s_{f}}=v_{max}^{2}$.

### 3.9. Time Window Constraints

**Figure 4:**
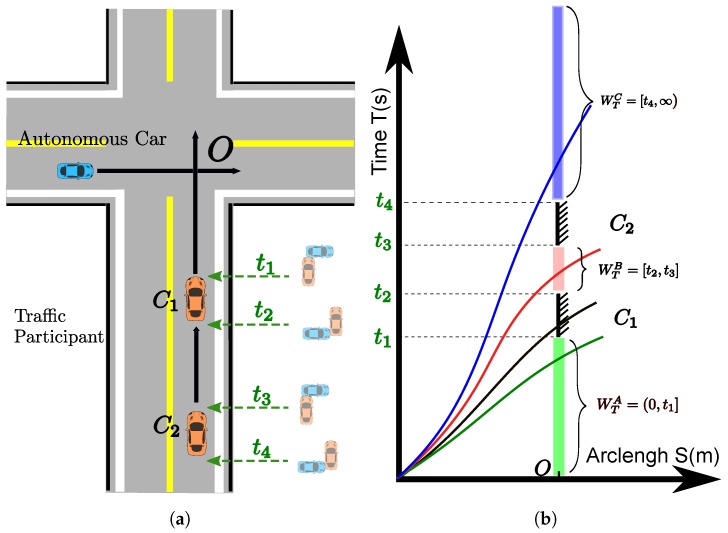
A cross scenario with moving vehicles. (**a**) A cross scenario without traffic lights. The blue car is the autonomous car. The orange cars ($C_{1}$, $C_{2}$) are the oncoming vehicles with prescribed speed profiles. (**b**) A S-T graph that describes different types of time windows and possible solutions to avoid moving vehicles. The *S* is the arc-length along the path of the autonomous car.

Time window constraints are represented as

(20) $$\begin{array}{c} t_{i}=T\left(s_{i}\right)\in W_{T}=\left(0,T_{U}\right], \end{array}$$

where $T\left(s_{i}\right)=\int_{0}^{s_{i}}\beta {\left(s\right)}^{-\frac{1}{2}}ds$ and $T_{U}>0$. The constraint ensures that if the car passes the station $s_{i}$ during the time window $W_{T}$, non-collision with other traffic participants is guaranteed. The time window, $W_{T}$, can be acquired efficiently from a collision detection algorithm such as [39] with predicted trajectories of traffic participants in the workspace-time space. This type of constraint is very useful for handling time-critical tasks such as dynamic obstacle avoidance at certain points, $s_{i}$, along the path, and for arriving at the destination within the given max time duration. If no time window information about dynamic obstacles is available, this constraint can be relaxed by setting $T_{U}=\infty$. In fact, there are three types of time windows when involving dynamic obstacles. Take the cross scenario without traffic lights in Figure 4a for example, the oncoming vehicles ($C_{1}$, $C_{2}$) are approaching the cross with predicted or prescribed speed profiles. They will occupy the station *O* during the time interval $\left[t_{1},t_{2}\right]$ and $\left[t_{3},t_{4}\right]$. These infeasible time intervals divide the feasible time window to three different pieces that have distinct forms. As shown in Figure 4b, the $W_{T}^{A}$ only has an upper boundary (see the green bar), and the $W_{T}^{B}$ owns both lower and upper boundaries (see the pink bar), and the $W_{T}^{C}$ has a lower boundary and an unlimited upper bound (see the blue bar). The complete feasible time window is an union of $W_{T}^{A}$, $W_{T}^{B}$ and $W_{T}^{C}$, which is non-convex since $W_{T}^{A}$ is convex and $W_{T}^{A}$, $W_{T}^{B}$ are non-convex. Inposing the combined time window seems straight-forward to do but will lead to a non-convex optimization problem, which makes our optimization problem hard to solve. In practice, a decision making system can rank the feasible time windows according to risks, energy to consume, or physical limits of vehicles, then select the best one to pass to the speed planning. For example, the black curve shows the previous solution without considering the oncoming vehicles. If the autonomous car does not regulate the speed, it will collide with the oncoming car $C_{1}$ during $\left[t_{1},t_{2}\right]$. By enforcing three different time windows constraints, three possible solution classes (green, red and blue curves in Figure 4b) are available. The time window size of the red curve class is very small. It means that it is very risky to go though this kind of time window. The blue curve class needs great control efforts to change the current state to satisfy the corresponding time window constraint. In the end, the green curve class becomes the best option since it needs minimum efforts to avoid the moving vehicles and has fairly low risks. By doing so, decision making can select a single time window constraint to enforce on the path with the help of other useful information. Hence, imposing a single time window that is convex becomes applicable while still keeping the problem in good structure. The type A time window expression $W_{T}^{A}$ is employed as the simplified and generalized convex time window constraint for the optimization shown as (20). For the time windows constraints like $W_{T}^{B}$ or $W_{T}^{C}$, we can pick $T_{U}\in W_{T}^{B}$ or $T_{U}\in W_{T}^{C}$ as the upper boundary to form the (20). Then a big coefficient for the smoothness objective can be used to “stretch” the travel time, which pushes the arrival time $t_{i}$ at the station $s_{i}$ to the upper boundary $T_{U}$. It is an indirect way to achieve the goal. The exact usage cases of this constraint can be found in Section 5.5 and Section 6.2.

### 3.10. Comfort Box Constraints

The comfort box constraint as another requirement of the ride comfort other than the smoothness, appears in a threshold form in the literature [7,9,11],

(21) $$\begin{array}{cc} ∥a_{i}^{\eta }∥ & \leq a_{c}^{\eta }\\ ∥a_{i}^{\tau }∥ & \leq a_{c}^{\tau }, \end{array}$$

which is a hard constraint. The $a_{c}^{\tau }$ is the threshold for the longitudinal accelerations and decelerations. The $a_{c}^{\eta }$ is the threshold for lateral accelerations. This box form of constraints ensures comfort at the cost of mobility. The mobility may dramatically drop if the comfort acceleration thresholds are set too conservatively. The feasible region for optimization is limited within a rectangle inside the friction circle if (21) is present, as shown in Figure 1. However, when an emergency occurs, the planner may have to violate the comfort constraint to leverage more mobility of the car to generate a safe speed profile by ignoring the comfort constraint temporally instead of failing by satisfying it. With a hard constraint presented in the problem, there is no way to reach this goal. Thus we employ a penalty method with slack variables to soften the comfort box constraint [40,41], which makes it a “*semi-hard*” constraint. If the original optimization problem was

(22) $$\begin{array}{cc} \underset{s}{\mathrm{minimize}} & J(s)\\ s.t. & c(s)\leq 0, \end{array}$$

an equivalent optimization problem using slack variables can be acquired as

(23) $$\begin{array}{cc} \underset{s}{\mathrm{minimize}} & J(s)+\lambda ∥\sigma ∥\\ s.t. & c(s)\leq \sigma \\ & 0\leq \sigma , \end{array}$$

where σ is the slack variable that represent the constraint violations, λ is the corresponding weight. When σ=0, the constraint is satisfied as a hard one. By doing so, we conserve the freedoms to explore full mobility of cars and capacity of breaking the comfort box constraint to recover the feasibility when necessary. The exact expression of the semi-hard constraint is shown in (24).

### 3.11. Overall Convex Optimization Problem Formulation

Finally, the complete speed planning optimization problem over the fixed path is posed, which incorporates the full set of constraints presented above as,

(24) $$\begin{array}{cc} \underset{\begin{array}{c} \alpha (s),\beta (s),u(s),\\ \sigma^{\tau }\left(s\right),\sigma^{\eta }\left(s\right) \end{array}}{\mathrm{minimize}} & J=\omega_{1}J_{T}+\omega_{2}J_{S}+\omega_{3}J_{V}\\ & +\lambda_{1}∥\sigma^{\tau }\left(s\right)∥+\lambda_{2}∥\sigma^{\eta }\left(s\right)∥\\ s.t. & (6),(8),(11),(17),(19),(20),\\ & ∥\alpha \left(s\right)∥\leq a_{c}^{\tau }+\sigma^{\tau }\left(s\right),\\ & ∥\frac{u^{\eta }\left(s\right)}{m}∥\leq a_{c}^{\eta }+\sigma^{\eta }\left(s\right),\\ & 0\leq \sigma^{\tau }\left(s\right),\\ & 0\leq \sigma^{\eta }\left(s\right), \end{array}$$

where ${\dot{r}}^{2}\left(s\right)={\left(r^{\prime }\left(s\right)\right)}^{2}\beta \left(s\right)$ and $\ddot{r}\left(s\right)=r^{\prime }\alpha \left(s\right)+r^{\prime \prime }\beta \left(s\right)$. Please note that $\alpha \left(s\right),\beta \left(s\right),u\left(s\right),\sigma^{\tau }\left(s\right),\sigma^{\eta }\left(s\right)$ are the decision variables to optimize. The parameters $\omega_{1},\omega_{2},\omega_{3},\lambda_{1},\lambda_{2}\in R_{+}$ are fixed in advance to suit the particular application objectives. When parameters $\lambda_{1},\lambda_{2}$ are both set to zeros, the $\sigma^{\tau }\left(s\right),\sigma^{\eta }\left(s\right)$ are degenerated to constants zeros and $a_{c}^{\tau },a_{c}^{\eta }$ are set to infinity, which means the comfort box constraint is relaxed. The problem formulation we presented can be demonstrated to be convex as follows. For these readers who are not familiar with convex optimization, we refer them to [40,42] for details.

- For the objectives, $J_{T}$ is an integral of a negative power function and is therefore convex. $J_{S}$ is an integral of a squared power of absolute value and is therefore convex. $J_{V}$ is an integral of an identity power of absolute value and is therefore convex. So are $∥\sigma^{\tau }∥$ and $∥\sigma^{\eta }∥$. As $\omega_{1}$, $\omega_{2}$, $\omega_{3}$, $\lambda_{1}$, $\lambda_{2}$ are all nonnegative, *J* as a nonnegative weighted sum of convex functions, is convex.
- For (6), the dynamics equality constraint is affine in α, β, *u* and is therefore convex. For equality constraints about decision variables (8), since the derivative is a linear operator, the relation between α and β is convex. For the inequality path constraint (17), $\beta (s_{i})$ is a sublevel set of convex set in the interval $\left[s_{m},s_{n}\right]$ and is thus convex. The equality and inequality constraints about boundary conditions (19) are linear constraints, thus convex. As the $T_{i}$ is an integral of a negative power function, therefore convex and $T_{U}$ is a fixed upper boundary, the time window inequality constraint (20) is a convex constraint.
- For the convex set constraint about the friction circle (11), the norm of *u* is convex, upper bounds are fixed and $v_{max}^{2}$ is fixed, so the control set constraint is the intersection of three convex sets and is therefore convex.
- The comfort box constraints with slack variables $\sigma^{\tau }$ and $\sigma^{\eta }$ are second-order cone constraints and convex.

Since the objectives are convex, equality constraints are affine and inequality constraints are convex, this optimization problem is convex [40]. The speed planning problem as stated is therefore an infinite-dimensional convex optimization problem.

## 4. Implementation

To solve the speed planning problem, we discretize the objectives, constraints and decision variables to form a finite dimensional approximated version of the original problem, which is known as direct transcriptions in optimal control. We consider N=200 segments along the path, thus N+1 discretised points for all these numerical experiments in Section 5. For one segment of the path, we assume constant acceleration, which is also used in [2,3]. According to (8), β(s) can be expressed as,

(25) $$\begin{array}{c} \beta \left(s\right)=\beta_{i}+\left(s-s_{i}\right)\left(\frac{\beta_{i+1}-\beta_{i}}{s_{i+1}-s_{i}}\right),s\in \left[s_{i},s_{i+1}\right]. \end{array}$$

It should be noted that a zero speed constraint will result in an infeasible optimization problem. In practice, two methods can be employed to avoid the singularity. The first one is pruning the path after the station where the speed is zero since the zero speed point is the switch point of the system. The speed of the pruned part of the path is set to zero or the pruned part of the path can be another speed planning problem with a zero start speed. The second method is to use a small speed value to approximate the zero. In this way, we can still evaluate the objectives, perform the optimization and get a solution. When speeds fall below a certain value (i.e., 0.02 m/s) in the solution, we can treat them as the zero speeds.

### 4.1. Discretization of $J_{T}$, $J_{S}$, and $J_{V}$

Substituting $\beta {\left(s\right)}^{-\frac{1}{2}}$ into (25) yields,

(26) $$\begin{array}{cc} J_{T_{i}} & =\int_{s_{i}}^{s_{i+1}}\beta {\left(s\right)}^{-\frac{1}{2}}ds\\ & =\int_{s_{i}}^{s_{i+1}}{\left(\beta_{i}+\left(s-s_{i}\right)\left(\frac{\beta_{i+1}-\beta_{i}}{s_{i+1}-s_{i}}\right)\right)}^{-\frac{1}{2}}ds\\ & =\frac{2\cdot \Delta s}{\sqrt{\beta_{i}}+\sqrt{\beta_{i+1}}}, \end{array}$$

where $\Delta s=s_{i+1}-s_{i}$ is a fixed arclength increment.

This integral can be approximated in the following form,

(27) $$\begin{array}{cc} J_{T} & =2\sum_{i=0}^{N-1}\frac{\Delta s}{\sqrt{\beta_{i}}+\sqrt{\beta_{i+1}}}. \end{array}$$

For the smoothness term, we use finite differences to approximate $\alpha^{\prime }\left(s\right)$, which yields

(28) $$\begin{array}{cc} J_{S} & =\int_{0}^{s_{f}}{∥\alpha^{\prime }\left(s\right)∥}^{2}ds\\ & =\sum_{i=0}^{N-1}{∥\frac{\alpha \left(s_{i+1}\right)-\alpha \left(s_{i}\right)}{\Delta s}∥}^{2}\Delta s. \end{array}$$

The $J_{V}$ can be directly represented by

(29) $$\begin{array}{c} J_{V}=\sum_{i=0}^{N-1}∥\beta \left(s_{i}\right)-v_{r}^{2}∥\Delta s. \end{array}$$

### 4.2. Discretization of $r^{\prime }\left(s\right)$ and $r^{\prime \prime }\left(s\right)$

The discrete form representations of constraints are straight-forward to define, with the exception of the dynamics constraint (6), which involves first and second order derivatives of r(s) with respect to the arclength *s*. We use finite differences to approximate $r^{\prime }\left(s\right)$,

(30) $$\begin{array}{c} r^{\prime }\left(s\right)=\frac{r\left(s_{i+1}\right)-r\left(s_{i}\right)}{s_{i+1}-s_{i}}, \end{array}$$

and a fourth-order Range-Kutta formula to approximate $r^{\prime \prime }\left(s\right)$,

(31) $$\begin{array}{c} r^{\prime \prime }\left(s\right)=\frac{r\left(s_{i-2}\right)-r\left(s_{i-1}\right)-r\left(s_{i}\right)+r\left(s_{i+1}\right)}{2\Delta s^{2}}. \end{array}$$

We model our problem using Convex.jl [43] , a convex optimization modeling framework in Julia, and solve it using a second-order cone programming solver from Gurobi [44].

## 5. Numerical Results

**Figure 5:**
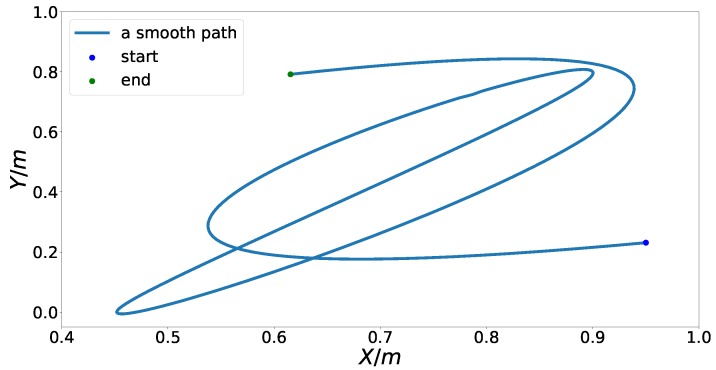
An example path from [3].

To evaluate the performance and capabilities of the proposed speed planning model, we use a curvy example path from [3], as shown in Figure 5, to conduct various challenging speed planning numerical experiments. To be fair, we implemented both our problem formulation and MTSOS in [3] in Julia [45] running on a PC with an Intel Xeon E3 processor at 2.8 GHz and 8 GB RAM in a Linux system and then compared our results with theirs to show the improvements and new capacities.

The used parameters are listed in Table 3. As they are a proof of concept experiment, these parameters do not match those of the real platforms. However, it does show the capacities of the speed planner from functional aspects. We will demonstrate the case studies using parameters from real platforms and dealing with real on-road driving scenarios in the next section.

As the friction circle constraint is the essence of the safety regarding vehicle dynamics, we enabled it for all the experiments below. We first run the MTSOS algorithm on the example path to generate the speed profile, accelerations and their distribution within the normalized friction circle as the baseline to compare with.

### 5.1. Smoothness

In this case, we show how the smoothness constraint of the our formulation affects the results and improve the performance. The initial speed $\sqrt{\beta (0)}$ of the car is a fixed point and assigned according to the current vehicle state in the optimization. In this case, we set the initial speed $\sqrt{\beta (0)}$ to 0 m/s and enable only friction circle constraint, time efficiency objective, smoothness objective by setting the parameters to

(32) $$\left\{\begin{array}{c} \omega_{1}=1,\omega_{2}\;\left(\mathrm{see}\;\mathrm{Figure}\;6\right),\;\omega_{3}=0\\ \mathrm{all}\;\mathrm{the}\;\mathrm{other}\;\mathrm{constraints}\;\mathrm{are}\;\mathrm{relaxed} \end{array}\right.$$

**Figure 6:**
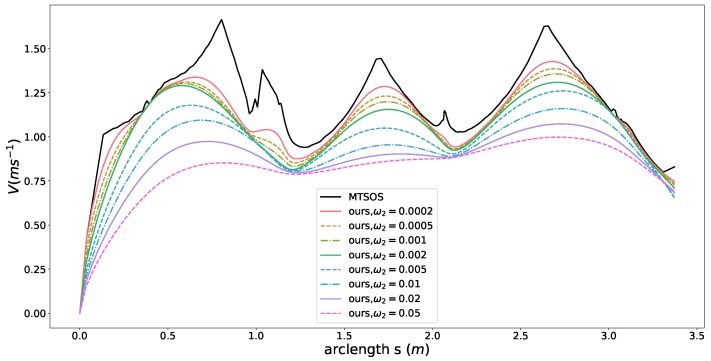
Speed planning results with different coefficients of the smoothness objective.

**Figure 7:**
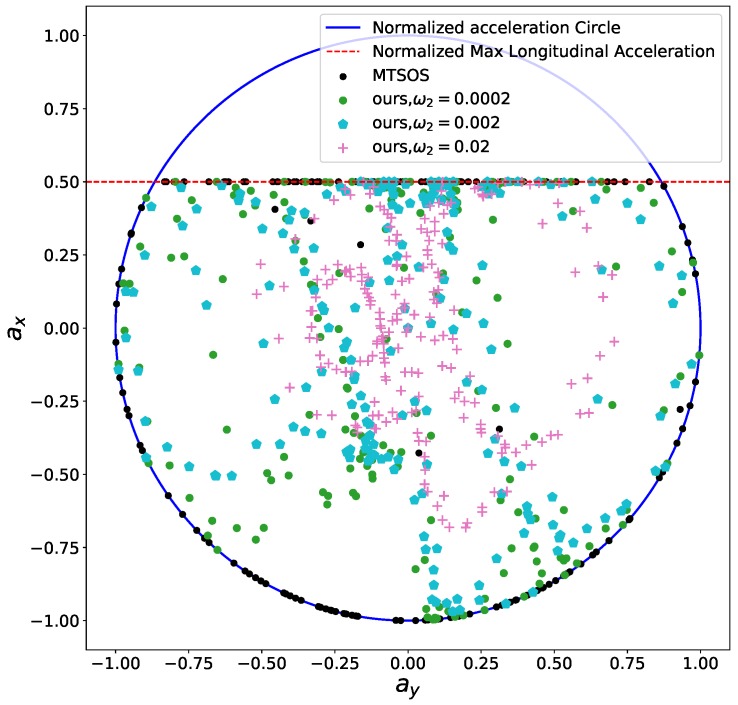
Friction circle for smoothness.

The other constraints are all relaxed or ignored to remove side effects and highlight the effects of the smoothness objective term. The black curve presented in Figure 6 represents the speed profile generated by MTSOS [3] with only time efficiency objective and friction circles constraints. The colored curves depict our results using different coefficients for the smoothness objective. Multiple cusps are observed in the MTSOS’s result, which definitely increases the difficulty of tracking such a speed profile for controllers. Overshooting and oscillation may happen when tracking a non-smooth speed profile such as the black one. Instead, our method generates way more smooth speed profile without cusps while still keeping time efficiency in mind. With small coefficients for smoothness, the resulting speed profiles tend to stay close to the most time-efficient speed profile (the black one) while still maintaining high order continuity. As coefficients of smoothness increase, flatter slopes of speed profiles are encouraged, thus smoother speed profiles are generated. With this structure in hand, our method offers a way to balance the time efficiency performance and smoothness performance according to specific application requirements when necessary. We also demonstrated control efforts distribution of MTSOS, ours with $\omega_{2}=0.0002$, $\omega_{2}=0.002$, and $\omega_{2}=0.02$ using a normalized friction circle (“g-g” diagram [46,47]), as seen in Figure 7. Since the MTSOS only considers the time efficiency, most of their acceleration points tend to stay close to the limits of accelerations. Ours, with the increase of smoothness coefficients, tend to lie around the center of the friction circle and reach the limits when necessary, which leads to a gentler control sequence. None of [3,4,8,10,48] show such high quality results as ours by taking both smoothness and time efficiency into consideration.

### 5.2. Boundary Condition Constraint

To demonstrate the capacity of boundary condition constraints, we carried out two set of experiments. In the first set of experiments, we compared the results with the following setting,

$$\begin{array}{c} \mathrm{MTSOS}\left\{\begin{array}{c} \mathrm{time}\;\mathrm{efficiency}\;\mathrm{objective}\\ \mathrm{free}\;\mathrm{final}\;\mathrm{speed}\\ \mathrm{friction}\;\mathrm{circle}\;\mathrm{constraint} \end{array}\right.\;\mathrm{ours}-A\left\{\begin{array}{c} \omega_{1}=1,\omega_{2}=0,\omega_{3}=0\\ \mathrm{final}\;\mathrm{speed}\;\mathrm{constraint}\;\beta (s_{f})=0\\ \mathrm{all}\;\mathrm{the}\;\mathrm{other}\;\mathrm{constraints}\;\mathrm{are}\;\mathrm{relaxed} \end{array}\right.\;\mathrm{ours}-B\left\{\begin{array}{c} \omega_{1}=1,\omega_{2}=0.002,\omega_{3}=0\\ \mathrm{final}\;\mathrm{speed}\;\mathrm{constraint}\;\beta (s_{f})=0\\ \mathrm{all}\;\mathrm{the}\;\mathrm{other}\;\mathrm{constraints}\;\mathrm{are}\;\mathrm{relaxed}. \end{array}\right. \end{array}$$

**Figure 8:**
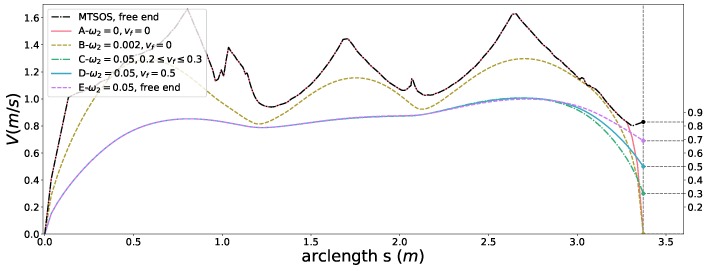
Speed planning results with different end boundary conditions.

The case ***A***, ***B*** in Figure 8 showed that our method is able to satisfy the final speed boundary condition while optimizing time efficiency (***A***) with a sharp slow-down slope or optimizing time efficiency and smoothness at the same time (***B***) with a flatter slow-down slope at the end. We conducted the second set of experiments with both time efficiency and smoothness objectives considered using same coefficients but with different type of boundary conditions,

$$\begin{array}{cc} \mathrm{ours}-C\left\{\begin{array}{c} \omega_{1}=1,\omega_{2}=0.05,\omega_{3}=0\\ \mathrm{inequality}\;\mathrm{constraint}:\;\\ \;\;\;\;0.2^{2}\leq \beta \left(s_{f}\right)\leq 0.3^{2}\\ \mathrm{all}\;\mathrm{the}\;\mathrm{other}\;\mathrm{constraints}\;\mathrm{are}\;\mathrm{relaxed} \end{array}\right.\;\mathrm{ours}-D\left\{\begin{array}{c} \omega_{1}=1,\omega_{2}=0.05,\omega_{3}=0\\ \mathrm{equality}\;\mathrm{constraint}:\;\\ \;\;\;\;\beta \left(s_{f}\right)=0.5^{2}\\ \mathrm{all}\;\mathrm{the}\;\mathrm{other}\;\mathrm{constraints}\;\mathrm{are}\;\mathrm{relaxed} \end{array}\right.\;\mathrm{ours}-E & \left\{\begin{array}{c} \omega_{1}=1,\omega_{2}=0.05,\omega_{3}=0\\ \mathrm{free}\;\mathrm{final}\;\mathrm{speed}\\ \mathrm{all}\;\mathrm{the}\;\mathrm{other}\;\mathrm{constraints}\;\mathrm{are}\;\mathrm{relaxed}. \end{array}\right. \end{array}$$

Without limiting the final speed, a speed profile such as ***E*** is generated, which is the optimal shape under the given objectives. By adding an equality constraint (***D***) and an inequality constraint (***C***) to the final speed, we observed notable differences of the last portion of the speed profile among these results. The last segments of the speed profile are adapted by the optimization to satisfy the given constraints. The other parts almost stay the same for case ***C***, ***D***, ***E*** due to global optimality. A similar phenomenon is observed between the results of MTSOS and case ***A*** in Figure 8. Only the part that needs to be adjusted is regulated. This is an appealing feature for speed tracking regarding temporal consistency of references and control stability. Since time efficiency is one of the objectives, it makes sense that the final speed of the case ***C*** reached the upper boundary at the end when given a feasible range.

Neither MTSOS [3] nor [11] can deal with this case due to the lack of corresponding constraints. Adding a similar constraint to the MTSOS requires re-arrangement of the problem and non-trivial, error-prone changes to their customized solver. Regarding the final speed constraint as a soft one like [11] cannot guarantee where and when the constraint is satisfied. Instead, our formulation and framework overcome above flaws.

### 5.3. Path Constraint

**Figure 9:**
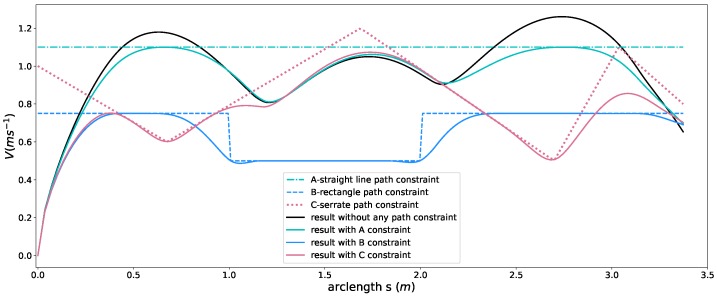
Speed planning results with different path constraints.

In this part, to show effects of path constraints, we conducted experiments with the friction circle constraint, time efficiency and smoothness objectives by $\omega_{1}=1$, $\omega_{2}=0.005$, $\omega_{3}=0$. For the sake of clarity, all the other hard constraints except path constraints are relaxed or ignored. For reference, a speed profile without any path constraint is generated using the given parameters (see the black curve in Figure 9), which can be thought of as the original speed profile before imposing the path constraints. Then we enforced three types of path constraints to show the capacity of our method,

- straight line shape (***A*** in Figure 9)
- rectangle shape (***B*** in Figure 9)
- serrated shape (***C*** in Figure 9)

as seen in Figure 9. The corresponding speed planning result is tagged using the same color with that of the path constraint. As shown in Figure 9, the original speed profile was deformed by optimization according to path constraints and all the resulting speed profiles stayed below the corresponding path constraints strictly while still keeping smooth. This provides a powerful tool for users to customize the speed profiles according to their needs while guaranteeing high quality of solutions.

### 5.4. IoD Task Constraints

**Figure 10:**
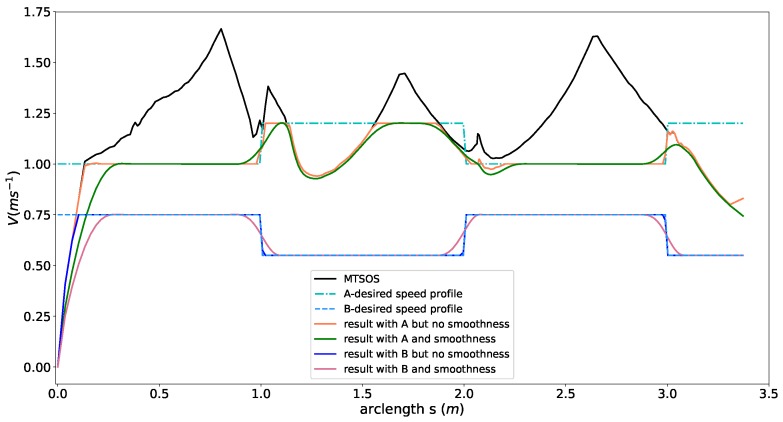
Speed planning results with desired speed in different shapes.

We evaluated effects of IoD task constraints using two different desired speed profiles (the dash-dot line ***A*** and the dash-dash line ***B*** in Figure 10) to show the behaviors of our planner. We first ran the MTSOS planner to generate the upper boundary of the speed profile for reference. For the desired speed profile ***A*** in Figure 10, we consider the time efficiency objective and IoD objective only by $\omega_{1}=1$, $\omega_{2}=0$, $\omega_{3}=10$ and relaxed all the other constraints to generate the speed profile, shown as the orange curve in Figure 10. The orange curve aligned well with the desired speed profile except for the part that the desired speed exceeds the limit of the friction circle. For the exceeding part, the orange curve stayed as close as possible to the desired speed but limited by the speed upper boundary constrained by the friction circle. This result uncovers the strong safety feature of our method. Moreover, taking the smoothness objective into consideration by making $\omega_{2}=0.1$, the quality of the speed profile is further improved (see the green curve in Figure 10). We also tested the IoD constraint against the totally feasible desired speed profile B using the same parameters setting with the previous experiment. The blue curve in Figure 10 depicted the planning result without considering smoothness. The resulting speed almost perfectly aligned with desired speed ***B***. Similarly, the quality of the speed profile was significantly improved by add the smoothness objective (see light red curve in Figure 10).

### 5.5. Time Window Constraint

**Figure 11:**
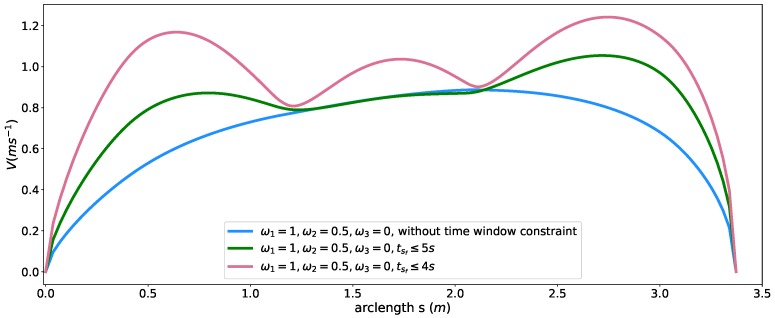
Speed planning results with time window constraints.

To reveal how the time window constraint affects the speed planning in our method, we first generate a baseline speed profile by considering only time efficiency and smoothness objectives with the following parameter setting, $\omega_{1}=1$, $\omega_{2}=0.5$, $\omega_{3}=0$, $\beta (s_{f})=0$. All the other hard constraints are relaxed. The result without time window constraints is shown as a blue curve in Figure 11. With a large coefficient for smoothness, the travel time at the end of the path reached 6.626 s. Please note that the time window constraint in (20) can be enforced on any point along the path. For simplicity, we picked the $s_{f}$ point as the place where imposing the constraint. We added the time window constraint by limiting the arriving time $T(s_{f})$ at the end of the path to $\left(0,T_{U}\right]$, where the $T_{U}=5$ s for case 1 and $T_{U}=4$ s for case 2 and solved them with respect to these constraints. The resulting speed profiles were shown as green and red curves for case 1 and case 2 in Figure 11, respectively. The travel time at $s_{f}$ are listed in Table 4 and both time constraints were satisfied according to the data. The original speed profile (blue one) were regulated to meet the time window requirements. The resulting speed profile was clearly above the original speed profile. This is a powerful tool that makes us able to control the time arriving at a certain point of the path by using a large coefficient for smoothness then enforcing the time window constraint to compress the travel time below the upper boundary of the given time window. In this way, we can easily “stretch” or “compress” the travel time for a fixed path. An example of “stretching” the travel time can be found in Section 6.2 case study.

### 5.6. Semi-Hard Comfort Box Constraint

To show the capacity of the semi-hard comfort box constraint, we conducted experiments with the following four different configurations,

$$\begin{array}{c} \mathrm{case}-A\left\{\begin{array}{c} \omega_{1}=1,\omega_{2}=0,\omega_{3}=0\\ \beta \left(s_{f}\right)=0,\lambda_{1}=2,\lambda_{2}=2 \end{array}\right.\;\mathrm{case}-B\left\{\begin{array}{c} \omega_{1}=1,\omega_{2}=0.05,\omega_{3}=0\\ \beta \left(s_{f}\right)=0,\lambda_{1}=2,\lambda_{2}=2 \end{array}\right.\\ \mathrm{case}-C\left\{\begin{array}{c} \omega_{1}=1,\omega_{2}=0,\omega_{3}=0\\ \beta \left(s_{f}\right)=0,\lambda_{1}=2,\lambda_{2}=2\\ t_{s_{f}}\leq 3.5s \end{array}\right.\;\mathrm{case}-D\left\{\begin{array}{c} \omega_{1}=1,\omega_{2}=0.05,\omega_{3}=0\\ \beta \left(s_{f}\right)=0,\lambda_{1}=2,\lambda_{2}=2\\ t_{s_{f}}\leq 3.5s. \end{array}\right. \end{array}$$

**Figure 12:**
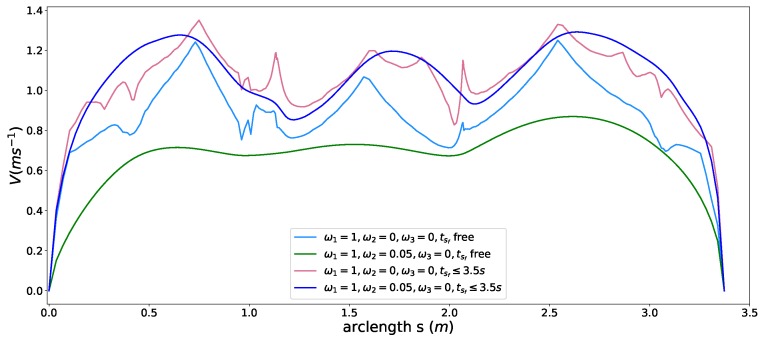
Speed profiles with semi-hard comfort box constraints.

**Figure 13:**
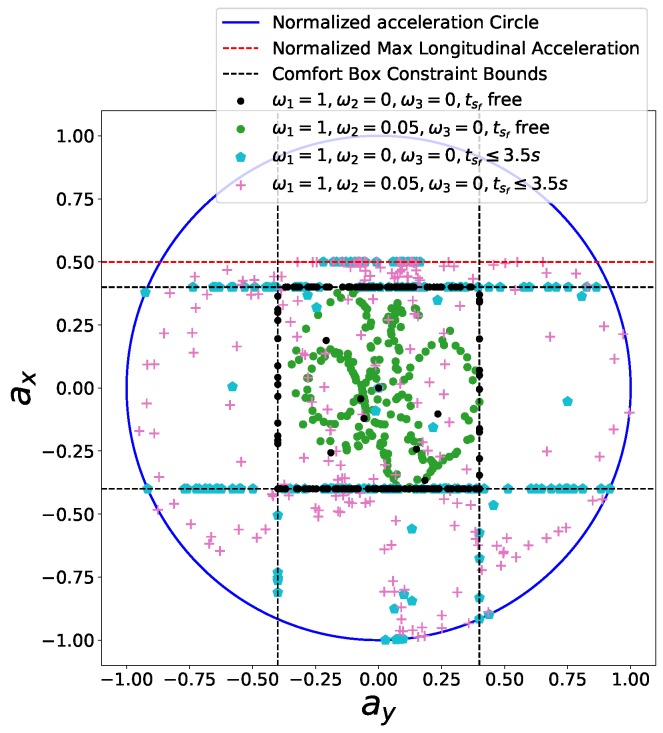
The g-g diagram of semi-hard comfort box constraints.

The comfort acceleration thresholds $a^{\tau }$ and $a^{\eta }$ are listed in Table 3. For case A, we only took the time efficiency objective into account and enable the comfort box constraints. The light blue curve in Figure 12 shows the resulting speed profile and the black dots in Figure 13 depict the resulting acceleration points distribution. Due to the presence of the time efficiency objective and limits of semi-hard comfort box constraints, most of the acceleration points tend to stay on the edge of box to achieve minimum travel time under such constraints. For case B, we add the smoothness objective in based on case A. The resulting speed profile is shown as the green curve in Figure 12, which is smoother than previous one. The rationale behind this is that the smoothness term encourages gentle control efforts to keep smooth transitions between states. Thus the acceleration points of case B more focused around the center of the friction circle while still staying inside of the box, shown as green dots in Figure 13. To demonstrate the “*semi-hard*” feature of our formulation, we imposes a time window constraint by making the final arriving time $t_{s_{f}}\leq 3.5$ s. With this constraint, the mobility constrained by the box region is no longer enough to achieve the required time efficiency. To get a solution that satisfies the time window constraint, the optimization has to exploit the region that is within the friction circle but outside of the box. The results of the acceleration points distribution of case 3 (see cyan pentagons in Figure 13) and case 4 (see pink pluses in Figure 13) proved our statements. The acceleration points were no longer limited within the box region. The corresponding speed curves were shown as the light red curve for case 3 and blue curve for case 4 in Figure 12. This nice feature distinguishes our method from existing speed planning methods such as [7,9,11] that regard comfort box constraints as hard ones like (21). Their methods guaranteed the ride comfort at the expense of losing potential mobility. Limiting accelerations to the comfort box region dramatically reduces the solution space of the speed planning problem, which may lead to no solution when one does exist in certain situation. Our method, instead, turns the comfort constraint to a semi-hard constraint by leveraging penalty functions and slack variables. More precisely, when the region limited by the box constraint is able to provide the needed mobility to satisfy other hard constraints, the slack variables are reduced to zero and the penalty functions have no effects on the optimization. The comfort box constraint is equivalent to a hard constraint. However, when the mobility provided by the box region is not enough to satisfy other hard constraints, slack variables increase and the penalty functions penalize the constraints violation. The comfort box constraint then is transferred to a soft constraint. By doing so, our method gives priority to the solution space in box region and leverages the outside region when necessary, which emphasizes comfort while keeping the solution space complete. To the best of our knowledge, none of the existing speed planning methods for autonomous driving has done this.

## 6. Case Study

In this section, we demonstrate three case studies to show how to combine constraints we present to solve distinct sets of speed planning problems raised in different real autonomous driving scenarios with parameters from the real platform like a Lincoln MKZ.

### 6.1. Speed Planning for Safe Stop

**Figure 14:**
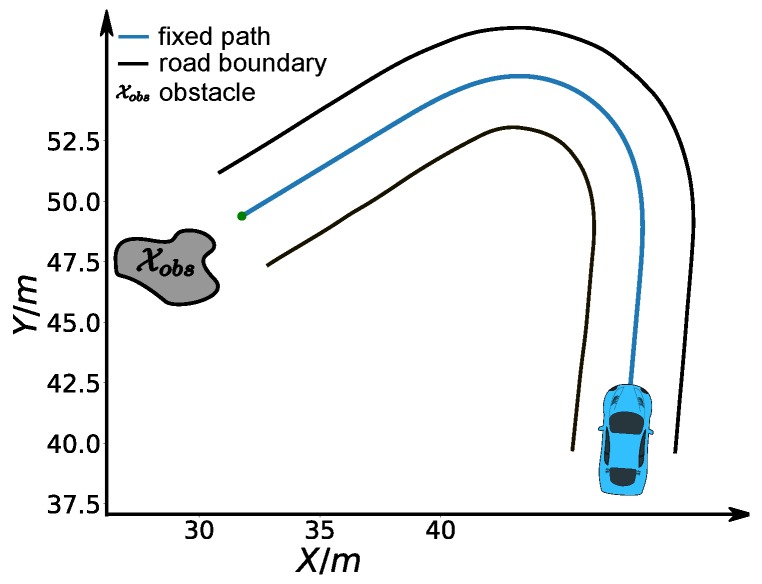
Safe stop scenario.

**Figure 15:**
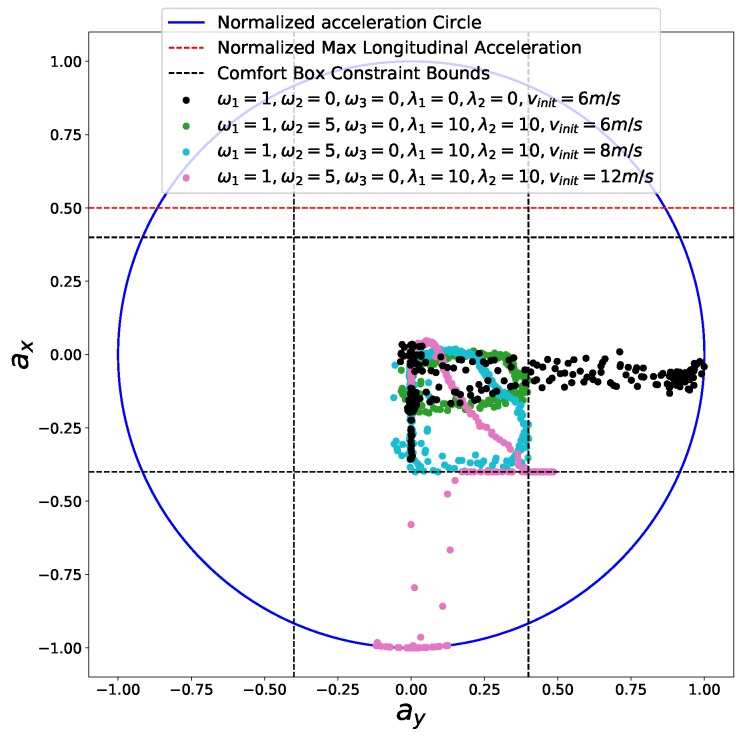
Friction circle for the safe stop scenario.

**Figure 16:**
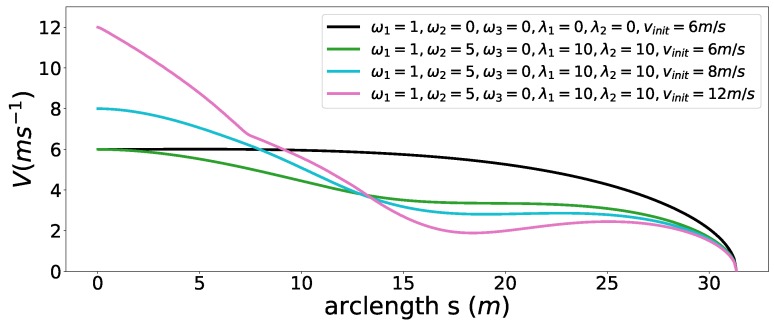
Speed profiles for the safe stop scenario.

First, we considered a cornering scenario (see Figure 14) with different entry speeds. At the end of the road, a static obstacle blocks the road and the car must stop safely in front of the obstacle. The comfort box constraints parameters used in this experiment are listed in Table 5. First, we perform speed planning that considers the time efficiency, smoothness objectives, friction circle and final speed constraints by making $\omega_{1}=1,\omega_{2}=5,\beta \left(s_{f}\right)=0$. The initial speed of the car is $v_{init}$ = 6 m/s. The semi-hard comfort box constraints were not taken into consideration in this one. The corresponding results are shown in Figure 15 and Figure 16 in black color. The second experiment was carried out using the same parameters. In addition, the semi-hard comfort box constraints were added by setting $\lambda_{1}=10$ and $\lambda_{2}=10$. The corresponding results are shown in green color. As depicted in Figure 15, when comfort box constraints were not presented, the optimization uses more control efforts when cornering and stopping for the sake of time efficiency. Once comfort box constraints were added, the control efforts were limited into the box region when mobility is enough to use. Next, we conducted the next two experiments using the same setting with that of the green one except two different initial speed $v_{init}$ = 8 m/s (cyan curves and dots) and $v_{init}$ = 12 m/s (pink curve and dots). As shown in Figure 15, when the initial speed increase to 8 m/s, the region constrained by comfort box was still able to provide enough mobility to stop at the end. Thus all the acceleration points stayed inside the box region. However, when the initial speed was increased dramatically to 12 m/s, the optimization had to use more control efforts to stop in the end. In consequence, the box constraints are “softened” and acceleration points went beyond the box region to guarantee a safe stop. With the comfort box constraint as a hard one, the method cannot get a solution in the last case.

### 6.2. Speed Planning Dealing with Jaywalking on a Curvy Road

**Figure 17:**
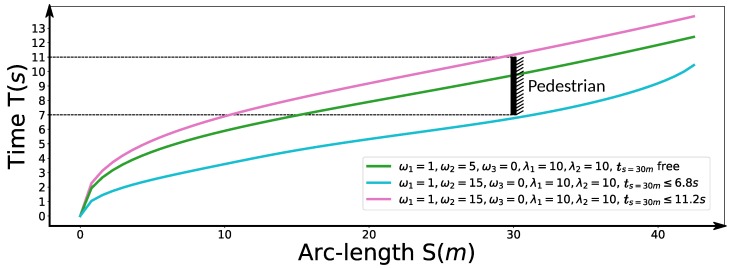
S-T graph for the jaywalking scenario.

**Figure 18:**
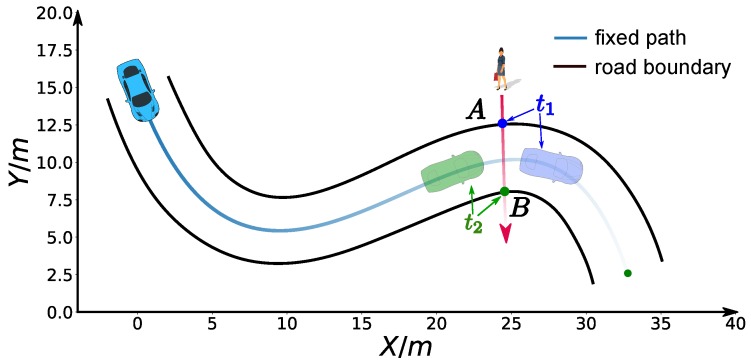
Jaywalking scenario.

**Figure 19:**
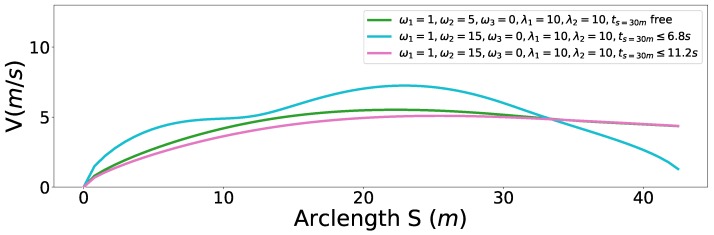
S-V graph for the jaywalking scenario.

**Figure 20:**
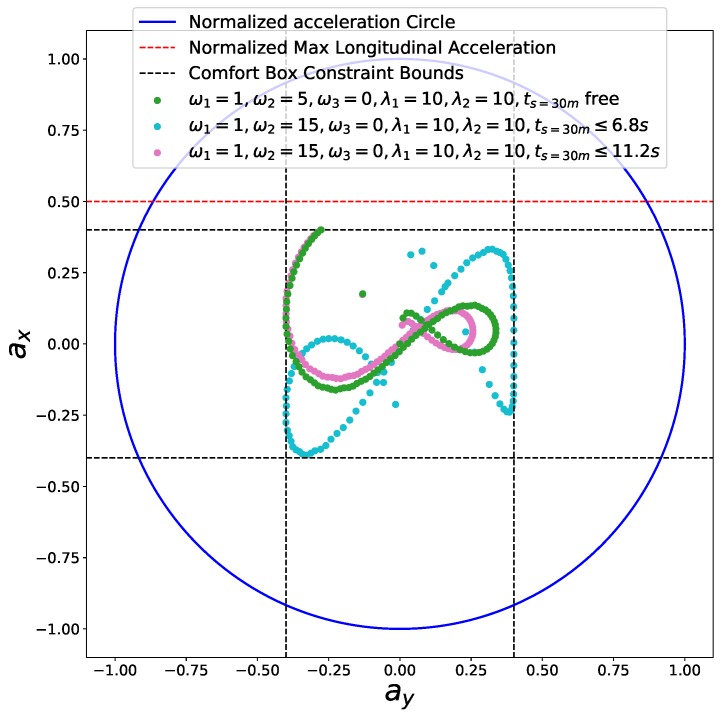
Friction circle for the jaywalking scenario.

Second, we considered a jaywalking scenario on a curvy road. The time window $[t_{1}$ = 7 s, $t_{2}$ = 11 s] that the pedestrian occupies the road at s=30 m is given by a dynamic obstacle prediction subsystem. As shown in the previous experiments, our method is able to stop at a specified point along the path. Here, we consider two advanced use cases to avoid the pedestrian safely without stop by manipulating the arrival time. Non-stop dynamic obstacle avoidance strategies may result in energy saving driving behavior or greatly reduced operation time in certain cases. As the pedestrian occupied the road between 7 s and 11 s at s=30 m along the path, if our car reaches s=30 m in the same time window, an accident may happen. Unfortunately, with the parameter setting $\omega_{1}=1,\omega_{2}\;=\;5,\omega_{3}\;=\;0,\lambda_{1}\;=\;10,\lambda_{2}=10$, our car will collide with the pedestrian, which is shown as the green curve in Figure 17. Two strategies can be employed to avoid this failure. The first involves passing the potential collision point before the pedestrian arrives point *A*, that is, $t_{s=30m}<=t_{1}$, which is shown as the blue car situation in Figure 18. The second involves passing the potential collision point just after the pedestrian passes point *B*, that is, $t_{s=30m}>=t_{2}$, which is shown as a green car situation in Figure 18. We solved this problem using both strategies. By making $\omega_{1}=1,\omega_{2}=15,\omega_{3}=0,\lambda_{1}=10,\lambda_{2}=10,t_{s=30m}<=6.8$ s, we solved the former case and the corresponding results are demonstrated in color cyan in Figure 17, Figure 19 and Figure 20. In practice, we may be not able to pass the barrier in time using the former strategy due to dynamics constraints of cars. The latter approach or a safe stop at a specified point along the path can be always employed to avoid collision. The latter approach is solved by setting $\omega_{1}\;=\;1,\omega_{2}\;=\;15,\omega_{3}\;=\;0,\lambda_{1}\;=\;10,\lambda_{2}\;=\;10,t_{s=30m}<=11.2$ s. The results are presented in color pink in Figure 17, Figure 19 and Figure 20. It should be noted that the second approach is an indirect method for avoiding collision in this scenario. We first stretch the time by increasing the coefficient $\omega_{2}$ from 5 to 15, then compress the arrival time by making $t_{s=30m}\leq 11.2$ s. The exact arrival time at s=30 m for three different cases are 10.656 s (green), 6.799 s (cyan), and 11.199 s (pink).

### 6.3. Speed Planning for Freeway Entrance Ramp Merging

**Figure 21:**
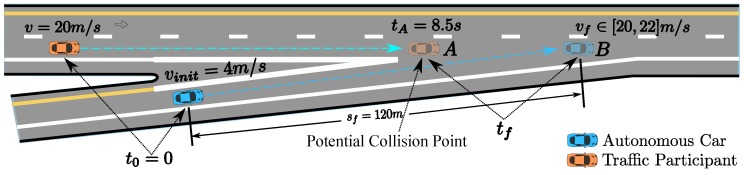
Freeway entrance ramp merging scenario.

**Figure 22:**
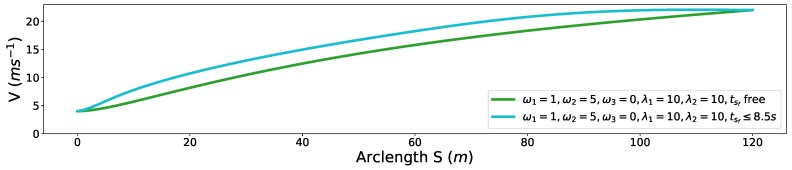
S-V graph for freeway entrance ramp merging.

**Figure 23:**
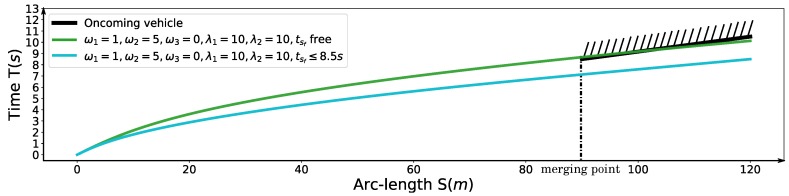
S-T graph for the freeway entrance ramp merging scenario.

Finally, we demonstrate a freeway entrance ramp merging scenario. The oncoming yellow car is driving in around 20 m/s. The arrival time $t_{A}=8.5$ s at merging point *A* in Figure 21 is given by the dynamic obstacle prediction or V2V communication module. The initial speed of the autonomous driving car is 4 m/s. With the parameter setting $\omega_{1}=1$, $\omega_{2}=5$, $\omega_{3}=0$, $\lambda_{1}=10$, $\lambda_{2}=10$, 20 m/s $\leq v_{f}\leq 22$ m/s, the arrival time $t_{s_{f}}$ at position *B* of the autonomous car provided by the optimization is 10.123 s. The related speed profile is shown as the green curve in Figure 22. The corresponding S-T graph is depicted in Figure 23 in green. The trajectory of the on-coming car is shown as the black curve in Figure 23. The scenario is designed such that the autonomous car would collide with the oncoming vehicle in the conflict zone if the oncoming car does not yield. To avoid the risk, we enforce a time window constraint at the end of the path, based on the previous parameter setting by making $t_{f}\leq 8.5$ s. In this way, the autonomous vehicle has already reached position *B* by the time the oncoming vehicle arrives position *A*, which also keeps a safe distance between the two vehicles. Further, the final speed of the autonomous car is constrained to be no less than that of the oncoming vehicle, which ensures that the safety is guaranteed. The corresponding solution is depicted by the cyan curve in Figure 22 and Figure 23. The exact arrival time at the end is 8.5 s.

In terms of the run-time performance, for 201 discretized points, the solving time range of our method is 0.05 s to 0.2 s with the Gurobi solver as the backend in Julia. For 100 discretized points, the solving time range is 0.03 s to 0.07 s. It is worth noting that the computation time may be greatly reduced if the algorithm is implemented in C++.

## 7. Conclusions

In this paper, we summarize and categorize the constraints needed to solve various speed planning problems in different scenarios as the *requirements* for speed planners design and *metrics* to measure the capacity of the existing speed planners for autonomous driving. Keeping these requirements and metrics in mind, we present a more general, complete, flexible speed planning mathematical model including time efficiency, friction circle, vehicle dynamics, smoothness, comfort, time window, boundary condition, speed deviations from desired speeds and path constraints for speed planning along a fixed path. The proposed formulation is able to deal with many more speed planning problems raised in different scenarios in both static and dynamic environments while providing high-quality, time-efficient, safety-guaranteed, dynamic-feasible solutions in one framework compared to existing methods. By considering the comfort box constraints as a semi-hard constraint and implementing it with slack variables and penalty functions in optimization, we emphasize comfort performance while guaranteeing fundamental motion safety without sacrificing the mobility of cars. We demonstrate that our problem preserves convexity with all these constraints added, therefore the global optimality is guaranteed. We conduct a range of numerical experiments to show how every constraint affects the speed planning results and showcase how our method can be used to solve speed planning problems by providing several challenging case studies in both static and dynamic environments. These results have depicted that the proposed method outperforms existing speed planners for autonomous driving in terms of constraint type covered, optimality, safety, mobility and flexibility.

Although our method is able to handle the dynamic obstacle with the time window constraint, it does rely on other modules to provide a single time window instead of the union of several time windows. It cannot handle multiple dynamic obstacles in optimization directly due to the non-convexity of the obstacle avoidance problem. In the future, nonconvex version of our problem formulation will be explored to specifically deal with multiple dynamic obstacles using the union of several time windows constraint in optimization directly. Since our problem is a multi-objective optimization problem, how to tune these coefficients systematically according to different autonomous driving applications will also be explored.

## Figures and Tables

**Table 1:** Constraints for speed planning.

Category	Constraint Name	Description	Property
Soft Constraints	Smoothness (S)	continuity of speed, acceleration and jerk over the path	performance
	Time Efficiency (TE)	time used by travelling along the path	performance
	IoD	integral of speed deviations	performance
Hard Constraints	Friction Circle (FC)	total force should be within the friction circle	safety
	Path Constraints (PC)	speed limits on path segments	safety
	Time Window (TW)	time window to reach a certain point on path	safety
	Boundary Condition (BC)	speed at the end of the path	safety & performance
Semi-hard Constraints	Comfort Box (CB)	comfort acceleration and deceleration bounds	performance

**Table 2:** Capacity of different speed planning methods.

Method	S	TE	IoD	FC	PC	TW	BC	CB	Optimality	Safety	Mobility	Flexibility
Li et al. [7]	✓	✗	✗	✗	✓	✗	✓	✓	✗	low	low	low
Gu et al. [8,9,10]	✓	✗	✗	✗	✓	✗	✓	✓	✗	medium	medium	medium
Dakibay et al. [4]	✗	✗	✗	✓	✓	✗	✓	✗	✗	medium	high	low
Liu et al. [11]	✓	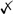	✓	✗	✗	✓	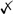	✓	local	medium	medium	medium
Lipp et al. [3]	✗	✓	✗	✓	✗	✗	✗	✗	global	low	high	low
Ours	✓	✓	✓	✓	✓	✓	✓	✓	global	high	high	high

**Mobility:** determined by how much mobility capacity of the vehicle the planner is able to leverage; **Optimality:** determined by whether the planner is able to identify an optimal solution in terms of its objective; **Flexibility:** determined by how many type of scenarios the planner is able to handle by only adjusting parameters without changing underlying problem formulation or problem structures; **Safety:** determined by four aspects, ability to stop in front of obstacles (BC) precisely, ability to deal with emergencies (FC), ability to impose task constraints like speed limits, and ability to handle dynamic obstacles (TW).

**Table 3:** Parameter Values.

Parameter	Description	Values	Unit
*m*	Mass of the car	0.1453	kg
μ	Friction coefficient	0.70	1
*g*	Acceleration of gravity	9.83	m/s${}^{2}$
$a_{c}^{\eta }$	Longitudinal acceleration threshold for comfort	0.4 μg	m/s${}^{2}$
$a_{c}^{\tau }$	Lateral acceleration threshold for comfort	0.4 μg	m/s${}^{2}$
$a_{max}^{\tau }$	Max. longitudinal acceleration of the car.	0.5 μg	m/s${}^{2}$
$v_{max}$	Max. speed of the car.	1.8	m/s

**Table 4:** Time Window Constraints.

Profile Figure 11	Coefficients	Time Window (s)	Travel Time at $s_{f}$ (s)
blue	$\omega_{1}=1,\omega_{2}=0.5$	free	6.626
green	$\omega_{1}=1,\omega_{2}=0.5$	$t_{s_{f}}\in \left(0,5\right]$	4.999
red	$\omega_{1}=1,\omega_{2}=0.5$	$t_{s_{f}}\in \left(0,4\right]$	4.000

**Table 5:** Parameter Values.

Parameter	Description	Values	Unit
*w*	Car width	2.45	m
*l*	Car length	4.9	m
wb	Car wheelbase	2.8448	m
tr	Car track	1.5748	m
*m*	Mass of the car.	1500.0	kg
μ	Friction coefficient	0.7	1
*g*	Acceleration of gravity	9.83	m/s${}^{2}$
$a_{c}^{\eta }$	Longitudinal acceleration threshold for comfort	0.4 μg	m/s${}^{2}$
$a_{c}^{\tau }$	Lateral acceleration threshold for comfort	0.4 μg	m/s${}^{2}$
$a_{max}^{\tau }$	Max. longitudinal acceleration of the car	0.5 μg	m/s${}^{2}$
$v_{max}$	Max. speed of the car	30	m/s
